# Synchronous Liver Abscess With Cholecystitis: A Rare Case of Initial Presentation of Gallbladder Cancer Posing a Diagnosis Challenge

**DOI:** 10.7759/cureus.36332

**Published:** 2023-03-18

**Authors:** Prabasha Weeraddana, Dinusha Dharmaratna, Mohammad A Ahmed-Khan, Niwanthi Weerasooriya, Susanna Josey, Oluwole Odujoko

**Affiliations:** 1 Internal Medicine, Danbury Hospital, Danbury, USA; 2 Pathology and Laboratory Medicine, Danbury Hospital, Danbury, USA

**Keywords:** intrahepatic abscess, gallbladder adenocarcinoma, severe sepsis, perforated cholecystitis, cholecystitis, liver abscess, gall bladder malignancy

## Abstract

Gallbladder carcinoma (GBC) is the most common of all biliary tract cancers. GBC is a multifactorial disease. Gallbladder dysplasia from any gallbladder inflammatory condition is one of the main risk factors for GBC. The late diagnosis of GBC is a major problem in its treatment. It is treated by radical resection and the prognosis is improved by adjuvant chemoradiation. We present a rare case of gall bladder cancer presenting as hepatic abscesses with severe sepsis. An 83-year-old male presented with progressive symptoms of shakiness, general weakness, vomiting, and profuse diarrhea. Lab work revealed deranged liver enzymes. Computed tomography (CT) and magnetic resonance cholangiopancreatography (MRCP) abdomen revealed intrahepatic abscesses contiguous with the gallbladder lumen through a gallbladder wall defect and cholecystitis of unknown chronicity. Subsequently, he underwent central hepatectomy and the pathology report of the sample as well as endoscopic retrograde cholangiopancreatography (ERCP) brushings revealed gallbladder adenocarcinoma. The case was complicated by biloma, acute renal failure, and the development of malignant ascites, and the patient died almost four months after the diagnosis of gallbladder cancer.

## Introduction

Gallbladder carcinoma (GBC) is a carcinoma of the biliary epithelium. The most common GBC is adenocarcinoma [1]. Advanced age, female gender, cholestasis, Salmonella species, and Helicobacter pylori infections are the most important risk factors for GBC [2]. The survival rate of GBC is very low, with the median survival time being one year. Radical resection is the treatment of GBC, and adjuvant therapies including chemotherapy and radiotherapy are also used [3]. GBC may present in a variety of ways, such as abdominal pain, vomiting, weight loss, jaundice, or palpable abdominal mass. Rarely it can present as a liver abscess. We present a case of gallbladder cancer presenting as hepatic abscesses with sepsis. The patient was initially treated with intravenous (IV) antibiotics for a prolonged duration. But after no improvement of abscess in follow-up imaging, the patient underwent hepatectomy, with cholecystectomy which revealed the diagnosis of adenocarcinoma of the gallbladder in the histopathology analysis.

## Case presentation

**Figure 1:**
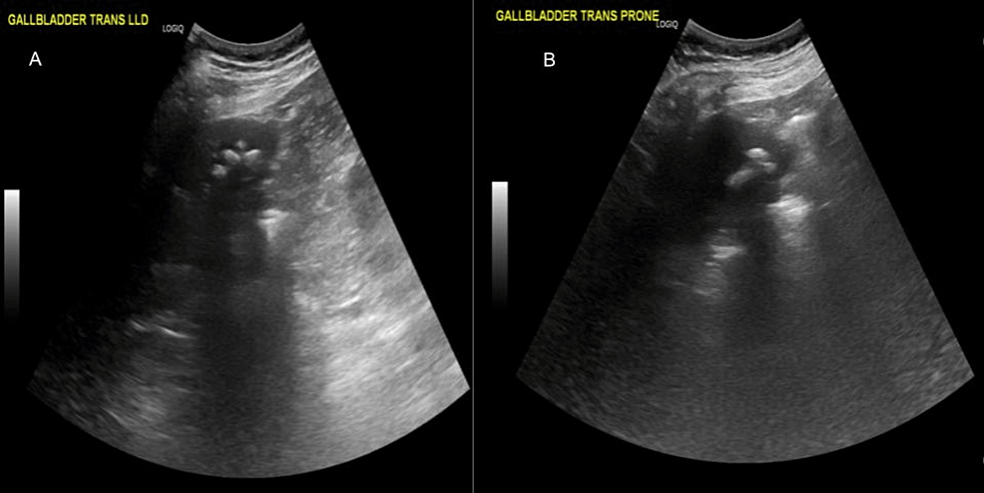
The ultrasound of the abdomen shows multiple stones within the gallbladder without evidence of acute cholecystitis.

**Figure 2:**
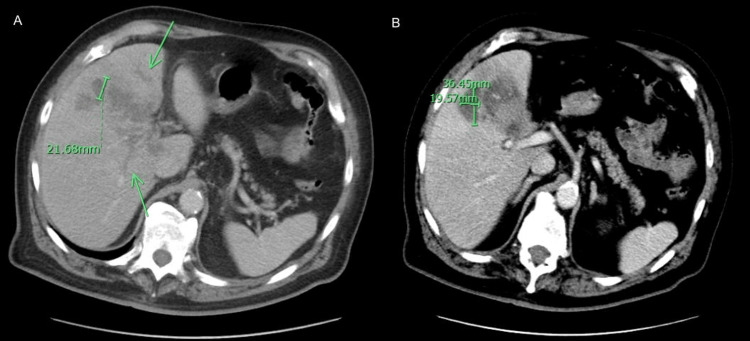
CT abdomen and pelvis with contrast shows low-density phlegmonous collection is noted scattered throughout the liver (green arrows in A), the largest measuring approximately 36.45mm (B). Computed tomography (CT)

**Figure 3:**
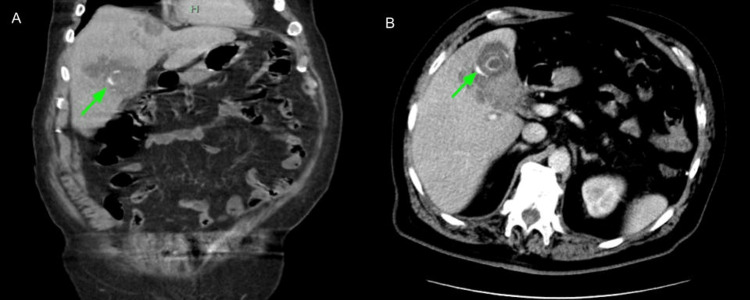
CT abdomen and pelvis with contrast shows multiple large gallstones within the gallbladder (green arrow in A - coronal view, B - axial view).

**Figure 4:**
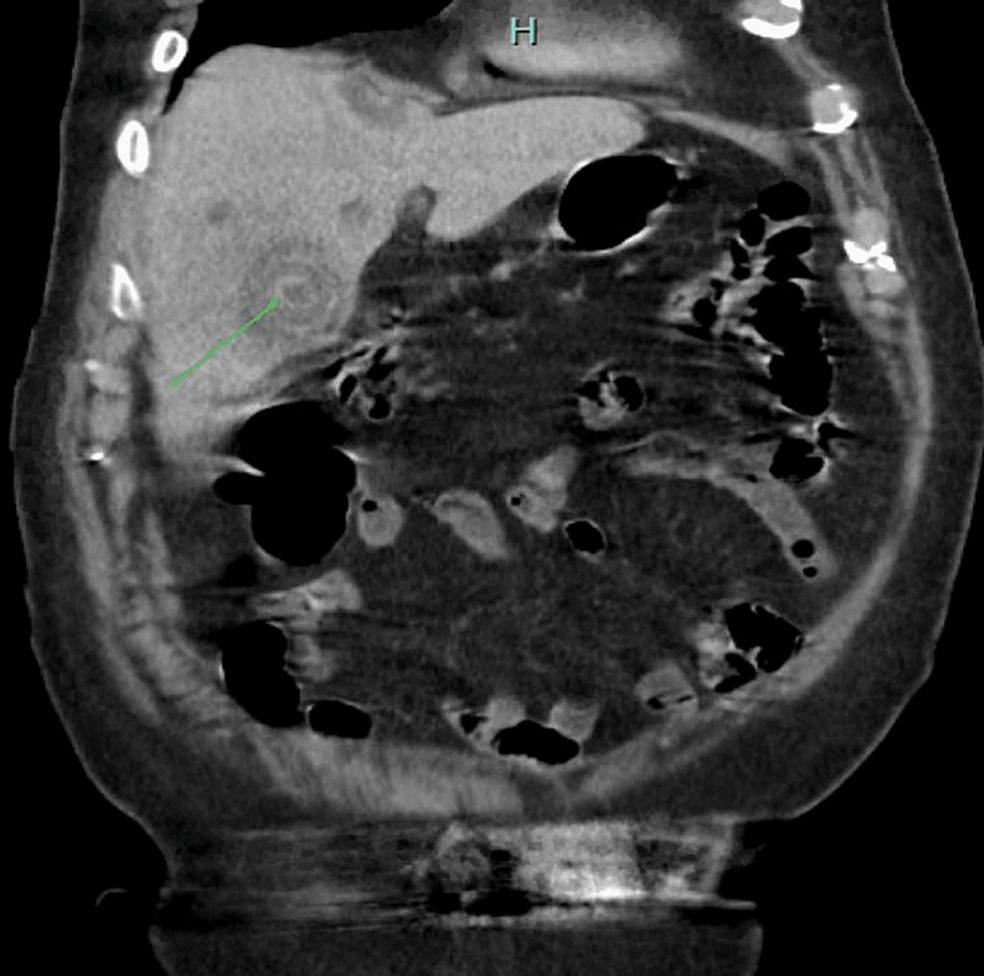
CT of the abdomen and pelvis with contrast in coronal view shows questionable mass versus sludge noted within the gallbladder lumen (green arrow).

An 83-year-old male with a past medical history of hypertension, type 2 diabetes, and hyperlipidemia presented to the emergency department with a complaint of progressive symptoms of shakiness, malaise, general weakness, and shortness of breath for the past two months. In addition, he developed several episodes of vomiting and profuse diarrhea over the past few days. The patient also revealed unintentional weight loss of 20 pounds in the past two months. He was a nonsmoker and denied using alcohol. There was no family history of malignancies. The patient returned from El Salvador two days prior to admission. On admission, the patient had a temperature of 102 F with tachycardia at 123; his abdomen was soft, non-tender, and not distended. There was no organomegaly. His lab work revealed a leukocyte count of 32.4 × 10^9^/L with neutrophil predominance, hemoglobin of 10.2 mg/dL, lactic acid of 1.7 mmol/L, creatinine of 1.94 mmol/L, and deranged liver enzymes with an aspartate transaminase (AST) of 95 U/L, an alanine transaminase (ALT) of 68 U/L, an alkaline phosphatase (ALP) of 208 U/L, and a gamma-glutamyl transferase (GGT) of 111 U/L. The total bilirubin was 1 mg/dL. The ultrasound of the abdomen showed multiple stones within the gallbladder (Figure 1). There was no evidence of acute cholecystitis. A contrast low-density phlegmonous collection was seen on the computed tomography (CT) of the abdomen and pelvis with contrast, with the largest measuring approximately 3.6 cm (Figures 2A, 2B). It also demonstrated multiple large gallstones within the gallbladder (Figures 3A, 3B). There was questionable mass versus sludge is noted within the gallbladder lumen (Figure 4). Findings were concerning for intrahepatic abscesses as well as significant gallbladder thickening.

**Figure 5:**
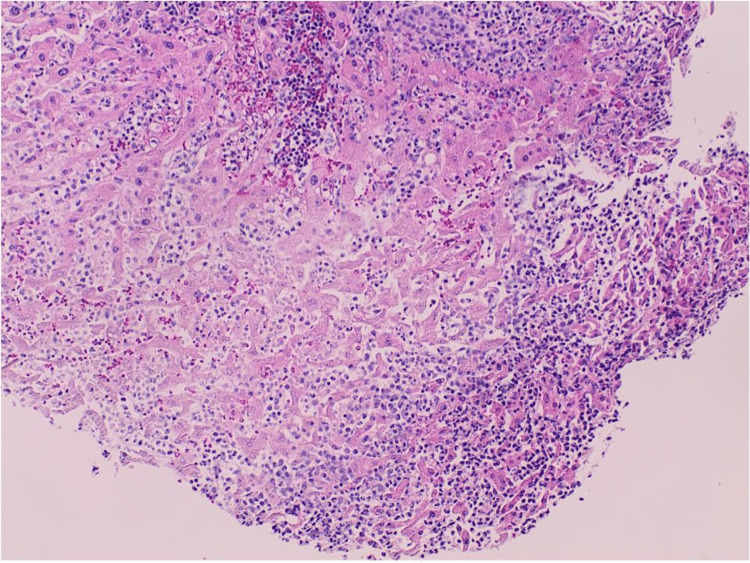
Liver abscess aspiration histology (H&E x 4) demonstrates liver tissue with normal hepatocytes at the upper half of the picture. There is marked infiltration of liver tissue in the lower half by neutrophilic cells with necrosis of the liver (liver abscess). Hematoxylin and eosin stain (H&E).

**Figure 6:**
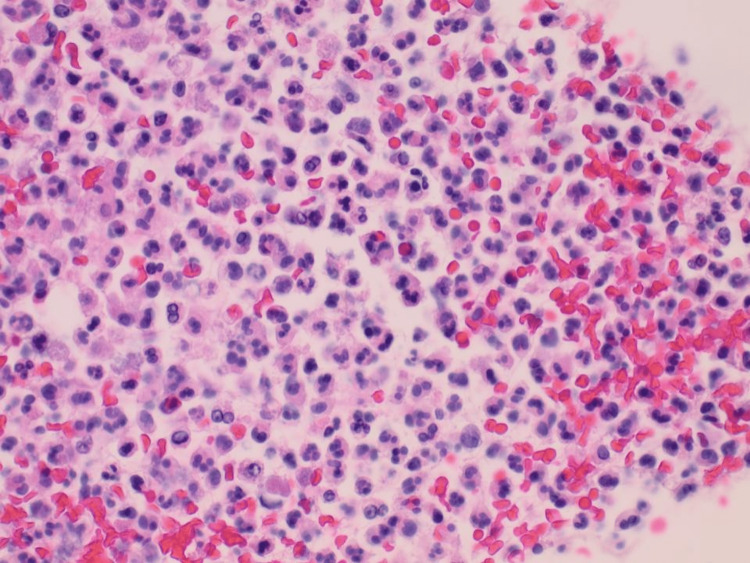
Liver abscess aspiration histology (H&E x 40) higher magnification shows the focus of necrotizing inflammation with neutrophils and some extracellular red cells.

**Figure 7:**
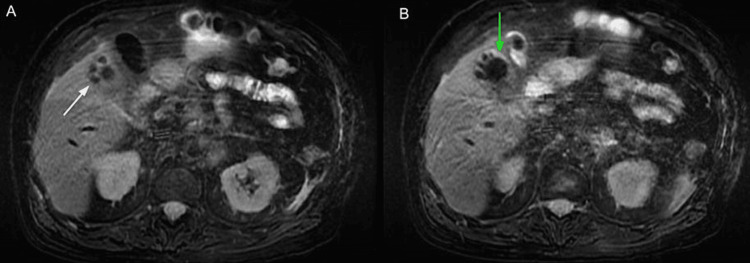
MRCP abdomen axial view shows gallbladder with multiple gallstones (white arrow in A) and inflamed gallbladder wall indicative of cholecystitis of unknown chronicity (green arrow in B). Magnetic resonance cholangiopancreatography (MRCP)

**Figure 8:**
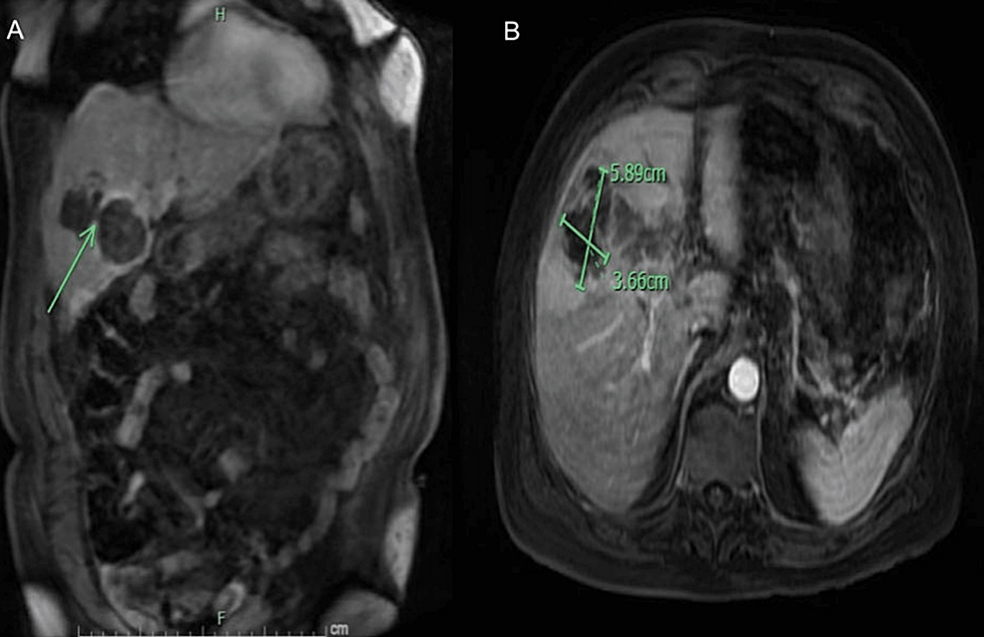
MRCP abdomen coronal view (A) 5.89 cm x 3.66 cm intrahepatic abscess in the liver that appeared to be contiguous with the gallbladder lumen through a gallbladder wall defect (green arrow), which was likely due to prior wall necrosis and rupture. (B) Axial view intrahepatic abscess.

**Figure 9:**
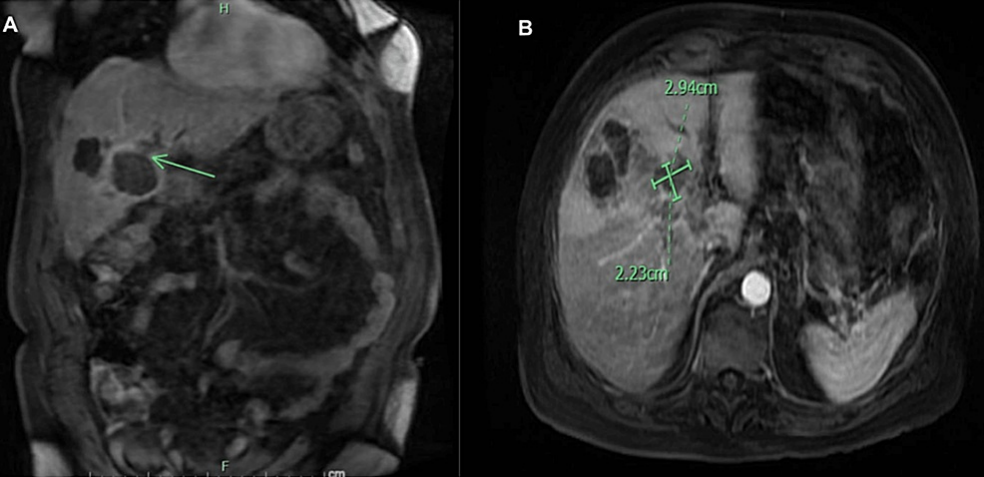
MRCP abdomen coronal view shows (A) 2.94 cm x 2.23 cm intrahepatic abscess that was also contiguous with the gallbladder (green arrow indicating communication between gallbladder wall and abscess). (B) Axial view of an intrahepatic abscess.

**Figure 10:**
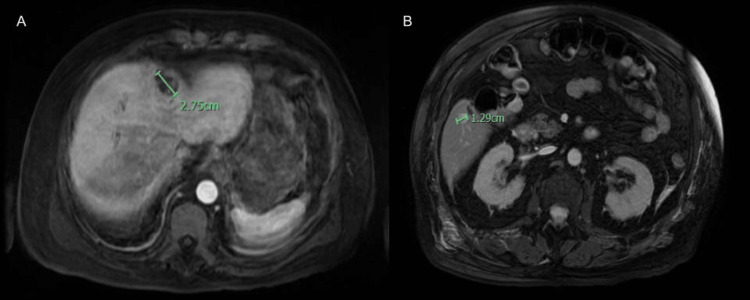
MRCP abdomen axial views show an additional two abscesses in the middle segment of the left hepatic lobe. (A) 2.25 cm abscess, (B) small 1.29 cm abscess.

He was admitted to the medical floors for the management of severe sepsis in the setting of a hepatic abscess. His blood cultures were positive for Escherichia coli. He was started on IV ceftriaxone and metronidazole. He had interventional radiology (IR)-guided aspiration of intrahepatic abscesses. The aspirate fluid also tested positive for E. coli. The cytology from the drainage fluid showed rare, degenerative, and poorly preserved cells (Figure 5). There was also the presence of acute inflammation, which was consistent with an abscess (Figure 6). To rule out malignancy, he underwent magnetic resonance cholangiopancreatography (MRCP), which revealed an inflamed gallbladder with multiple gallstones indicative of cholecystitis of unknown chronicity (Figures 7A, 7B). There was a 5.9 cm intrahepatic abscess in the liver that appeared to be contiguous with the gallbladder lumen through a gallbladder wall defect, which was likely due to prior wall necrosis and rupture (Figures 8A, 8B). There was also a separate 2.9 cm intrahepatic abscess that was also contiguous with the gallbladder (Figures 9A, 9B). There were two additional distant biliary intrahepatic abscesses in the middle segment of the left hepatic lobe (Figure 10). No common bile duct (CBD) dilation was noted.

 

**Figure 11:**
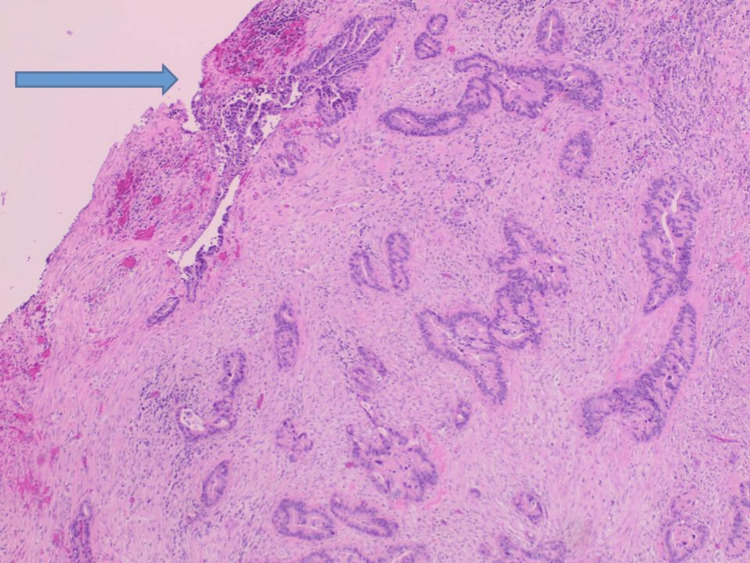
Histology (H&E 4) of surgically resected gallbladder revealed effacement of its architecture due to the proliferation of malignant epithelial cells that form glands in most areas into the underlying muscularis. The surface shows a dysplastic precursor epithelial lining of the gallbladder (blue arrow).

**Figure 12:**
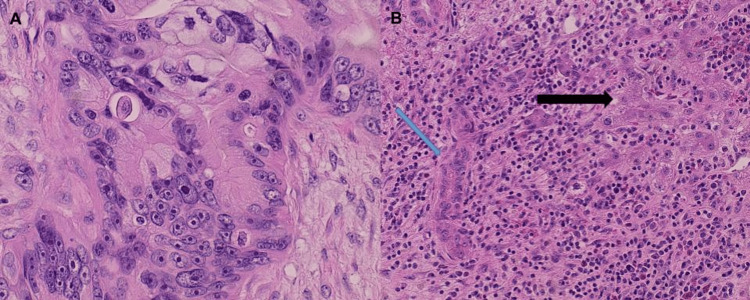
Histology of liver resection. (A) (H&E x 40) malignant cells with marked cellular and nuclear pleomorphism with prominent nucleoli. (B) (H&E x 20) Malignant glands (blue arrow) infiltrating into normal liver hepatocytes (black arrow) with surrounding brisk host inflammatory response.

He was discharged with IV Ceftriaxone 2g daily with oral metronidazole for four to six weeks. His follow-up CT scan of the abdomen pelvis shows persistent intrahepatic abscess. Therefore, he subsequently underwent an exploratory laparotomy, intraoperative ultrasound, a central hepatectomy, a cholecystectomy, and drainage of the hepatic abscess by hepatobiliary surgery. The surgery was complicated by a post-op biliary leak requiring endoscopic retrograde cholangiopancreatography (ERCP) with sphincterotomy and stent placement for significant biliary stricture in the upper third of the main bile duct. The pathology report from the surgical resection and ERCP brushings revealed gallbladder adenocarcinoma with moderately differentiated transmural invasion into the overlying liver parenchyma and secondary abscess formation. Perineural invasion was positive (Figures 11, 12A, 12B). The surgical margin was focally positive for carcinoma. Due to the newly diagnosed gallbladder cancer, the patient was referred to oncology for further management of the cancer. Radiation therapy was recommended, but he refused. The patient had an Eastern Cooperative Oncology Group (ECOG) poor performance status; therefore, he was not a good candidate for chemotherapy. Subsequently, he had another ERCP with stent removal and placement of left and right metal intrahepatic stents, and IR drainage of a biloma with pigtail catheter placement. Later, he developed malignant ascites. For that, he underwent the placement of a tunneled intraperitoneal catheter for long-term ascites drainage.

His case was complicated by the development of an acute injury, requiring renal replacement therapy. The patient could not tolerate hemodialysis due to symptomatic hypotension. The patient expressed his desire to stop all treatments and go home with a comfort measurement. Therefore, he was placed on hospice care and comfort measures. But unfortunately, he passed away on the day of transition to comfort measures only (CMO), about four months after his diagnosis of gallbladder cancer.

## Discussion

GBC is the most prevalent type of biliary tract cancer, which also encompasses malignancies of the intrahepatic and extrahepatic bile ducts. GBC develops from the mucosal lining of the gallbladder [4]. Adenocarcinomas account for the majority (80%-95%) of GBC cases reported. In developed countries, this cancer is uncommon, although it is more prevalent in several underdeveloped nations [5]. The worldwide prevalence of gallbladder cancer is less than two per 100,000 individuals [6].

Patients with GBC have a median survival duration of less than one year, an overall survival rate (OS) of roughly 17.8%-21.7%, and a five-year OS of only 5% [3]. Before becoming an invasive malignancy, gallbladder cancer develops through a series of significant events. The multistage pathogenesis of GBC begins with chronic cholecystitis [5]. This inflammation of the gallbladder then progresses to dysplasia and carcinoma in situ (with more than 90% of patients with GBC exhibiting this trend [7]). GBC is also associated with numerous genetic changes [7]. The ERBB pathway was discovered, by exome sequencing, to be the most dysregulated pathway in gallbladder cancer tissue [5].

It’s rare for GBC to present as a liver abscess with severe sepsis. In our case, the patient presented with vague symptoms of shakiness, malaise, and loose stools. Imaging studies (CT and MRCP of the abdomen) of our patient indicated cholecystitis with a ruptured gallbladder leading to liver abscess. His blood cultures as well as abscess fluid grew E. coli bacteria. Therefore, the patient was initially managed with IV antibiotics. But as the liver abscesses were not responding to antibiotics, he underwent surgical resection which prompted to detection of adenocarcinoma of the gall bladder. This rare presentation posed a diagnostic challenge [8,9].

GBC symptoms can be difficult to distinguish from benign diseases since they match those of biliary colic. Weight loss and jaundice are frequently linked to more advanced diseases, as is the case with other bile system malignancies [2]. Due to the absence of a peritoneal lining on the hepatic surface, tumor spread into the liver parenchyma is relatively common [2]. Carcino-embryonic antigen (CEA) and carbohydrate antigen 19-9 are the only two tumor markers most often elevated in advanced stages of GBC, but they have a poor degree of specificity. Macrophage galactose-specific lectin-2 binding protein (Mac-2BP), with variable sensitivity and specificity, is also found to be associated with GBC [5]. Many cases of GBC are discovered incidentally during Cholecystectomy as in our case. Due to the increase in the number of laparoscopic cholecystectomies performed, the number of incidentally discovered GBCs has also increased [10].

Female sex, advanced age, cholelithiasis or other benign gallbladder pathology, chronic infection with Salmonella species or Helicobacter pylori, porcelain gallbladder, gallbladder polyps, and obesity are the most important risk factors for developing GBC. A family history of cholelithiasis, chronic cholecystitis, high parity, smoking, exposure to chemicals (benzene), carbohydrate-rich food intake, and chronic bowel problems are secondary risk factors for the development of GBC [2]. We could not find any of the above risk factors in our case.

GBC affects women globally 2-3 times more frequently than men, however, bias varies widely over the world, especially in high-prevalence regions of GBC [11]. According to a paper by Pandey et al., people with the A+ and AB+ blood types had a higher prevalence of gallbladder cancer, however, the cause of this is yet unknown [12].

Surgery, chemotherapy, radiation, and other immunotherapies are all used to treat GBC. Despite GBC's high invasiveness and metastasis, surgical excision is still the preferred course of action. However, the majority of GBC patients are already at an advanced stage when they are discovered, which reduces the possibility of radical resection. Even at a moderate level, the prognosis for patients who have radical resection is quite poor [3]. Patients with stage T4 GBC may not benefit from surgical resection, according to Groot et al. [13]. For patients with advanced GBC, Mao et al. reported that the “surgery + chemotherapy” treatment strategy offered significant survival advantages [3]. Unfortunately, in our case, the patient had an Eastern Cooperative Oncology Group (ECOG) poor performance status and was not a suitable candidate for chemotherapy.

We present the case of an 83-year-old male who presented with a complaint of shakiness, malaise, shortness of breath, vomiting, and profuse diarrhea that was initially managed as severe sepsis secondary to liver abscess. Later he was diagnosed with gallbladder adenocarcinoma perforating into liver parenchyma causing the liver abscesses. The patient refused radiation therapy and chemotherapy could not be administered because of poor performance status. The patient's condition deteriorated over time with the development of malignant ascites, acute kidney injury, and biloma. He decided to transition to CMO and died four months after being diagnosed with GBC.

## Conclusions

Gallbladder cancers are highly aggressive tumors with poor prognoses. The presentation of gallbladder cancer as an intrahepatic abscess is a rare manifestation. Because its non-specific clinical presentation and radiological findings make a diagnostic challenge. Thus, GBC should be a differential diagnosis in the case of an intrahepatic abscess that does not respond to antibiotics. Late diagnosis can lead to curative resection and adjuvant chemotherapy difficulty increasing mortality.

## References

[1] Vaittinen E (1970). Carcinoma of the gall-bladder. A study of 390 cases diagnosed in Finland 1953-1967. Ann Chir Gynaecol Fenn Suppl.

[2] Hickman L, Contreras C (2019). Gallbladder cancer: Diagnosis, surgical management, and adjuvant therapies. Surg Clin North Am.

[3] Mao W, Deng F, Wang D, Gao L, Shi X (2020). Treatment of advanced gallbladder cancer: a SEER-based study. Cancer Med.

[4] Schmidt MA, Marcano-Bonilla L, Roberts LR (2019). Gallbladder cancer: epidemiology and genetic risk associations. Chin Clin Oncol.

[5] Sharma A, Sharma KL, Gupta A, Yadav A, Kumar A (2017). Gallbladder cancer epidemiology, pathogenesis and molecular genetics: recent update. World J Gastroenterol.

[6] Shaffer EA (2008). Gallbladder cancer: the basics. Gastroenterol Hepatol (N Y).

[7] J. Albores-Saavedra, A. Alcántra-Vazquez, H. Cruz-Ortiz (1980). The precursor lesions of invasive gallbladder carcinoma. Hyperplasia, atypical hyperplasia and carcinoma in situ. Cancer.

[8] Singla SL, Garg P, Tahlan RN (1998). Gall bladder carcinoma presenting as liver abscess. Indian J Gastroenterol.

[9] Zhou Zhou, Qian MD; Schafir, Alexander MD (2018). An unusual presentation of primary gallbladder carcinoma: 1361. Am J Gastroenterol.

[10] Wernberg JA, Lucarelli DD (2014). Gallbladder cancer. Surg Clin North Am.

[11] Randi G, Franceschi S, La Vecchia C (2006). Gallbladder cancer worldwide: geographical distribution and risk factors. Int J Cancer.

[12] Pandey M, Khatri AK, Dubey SS, Gautam A, Shukla VK (1995). Erythrocyte membrane fatty acid profile in patients with primary carcinoma of the gallbladder. J Surg Oncol.

[13] Groot Koerkamp B, Fong Y (2014). Outcomes in biliary malignancy. J Surg Oncol.

